# Combining nanotechnology with monoclonal antibody drugs for rheumatoid arthritis treatments

**DOI:** 10.1186/s12951-023-01857-8

**Published:** 2023-03-25

**Authors:** Xiao-Kai Chi, Xiao-Ling Xu, Bang-Yao Chen, Jin Su, Yong-Zhong Du

**Affiliations:** 1 College of Pharmacy, Jiamusi University, 258 Xuefu Road, Jiamusi, 154007 China; 2 Shulan International Medical College, Zhejiang Shuren University), 8 Shuren Street, Hangzhou, 310015 China; 3 Institute of Pharmaceutics, College of Pharmaceutical Sciences, Zhejiang University, 866 Yu-Hang-Tang Road, Hangzhou, 310058 China

**Keywords:** Rheumatoid arthritis, Antibody drugs, Drug delivery nanosystem

## Abstract

Rheumatoid arthritis (RA) is a systemic immune disease characterized by synovial inflammation. Patients with RA commonly experience significant damage to their hand and foot joints, which can lead to joint deformities and even disability. Traditional treatments have several clinical drawbacks, including unclear pharmacological mechanisms and serious side effects. However, the emergence of antibody drugs offers a promising approach to overcome these limitations by specifically targeting interleukin-1 (IL-1), interleukin-6 (IL-6), tumor necrosis factor-alpha (TNF-α), and other cytokines that are closely related to the onset of RA. This approach reduces the incidence of adverse effects and contributes to significant therapeutic outcomes. Furthermore, combining these antibody drugs with drug delivery nanosystems (DDSs) can improve their tissue accumulation and bioavailability.Herein, we provide a summary of the pathogenesis of RA, the available antibody drugs and DDSs that improve the efficacy of these drugs. However, several challenges need to be addressed in their clinical applications, including patient compliance, stability, immunogenicity, immunosupression, target and synergistic effects. We propose strategies to overcome these limitations. In summary, we are optimistic about the prospects of treating RA with antibody drugs, given their specific targeting mechanisms and the potential benefits of combining them with DDSs.

## Introduction

### Rheumatoid arthritis

Rheumatoid arthritis (RA) is an autoimmune disease, characterized by chronic synovitis and joint injury. In severe cases, bone erosion can occur, leading to loss of joint function and even disability. This condition can seriously affect the patient’s quality of life and longevity. The main clinical symptoms in patients with RA are morning stiffness, joint swelling, and pain, which can also affect extra-articular organs. In addition, patients typically exhibit elevated indices, such as rheumatoid factor (RF), anti-citrullinated protein/peptide antibody (ACPA), and other characteristic indicators [1]. Globally, the incidence of RA in the population is approximately 0.5%, and it is related to sex. The morbidity rate is approximately 3.6% in adult women and 1.7% in men, with women being affected 2–3 times more often than men [2–4].

Recently, the pathogenesis of RA remains inconclusive. It is generally agreed that multifactor, such as genetic and environmental factors, synergistically disturbs the immune system, resulting in unnecessary immune responses. Autoreactive T lymphocytes and B lymphocytes promote the immune response against autoantigens, which is considered the central driving factor of the disease. T lymphocytes can differentiate into a variety of helper T-cell (Th cell) subsets and secrete abundant inflammatory cytokines, such as tumor necrosis factor-alpha (TNF-α), interleukin-1 (IL-1), and interleukin-17 (IL-17), which infiltrate, aggregate, and invade the synovium of joints, resulting in inflammation [5, 6].

B lymphocytes produce antibodies known as RFs, which also mediate the occurrence of inflammation. Fibroblast-like synoviocytes (FLSs), the primary cells of the synovium, can release cytokines and chemokines and exhibit obvious invasion in the synovium. Therefore, FLSs are considered the fundamental participants in synovitis. In addition, FLSs can produce matrix metalloproteinases (MMPS), which can disrupt cartilage or joint damage in patients [7, 8, 12].

**Fig. 1:**
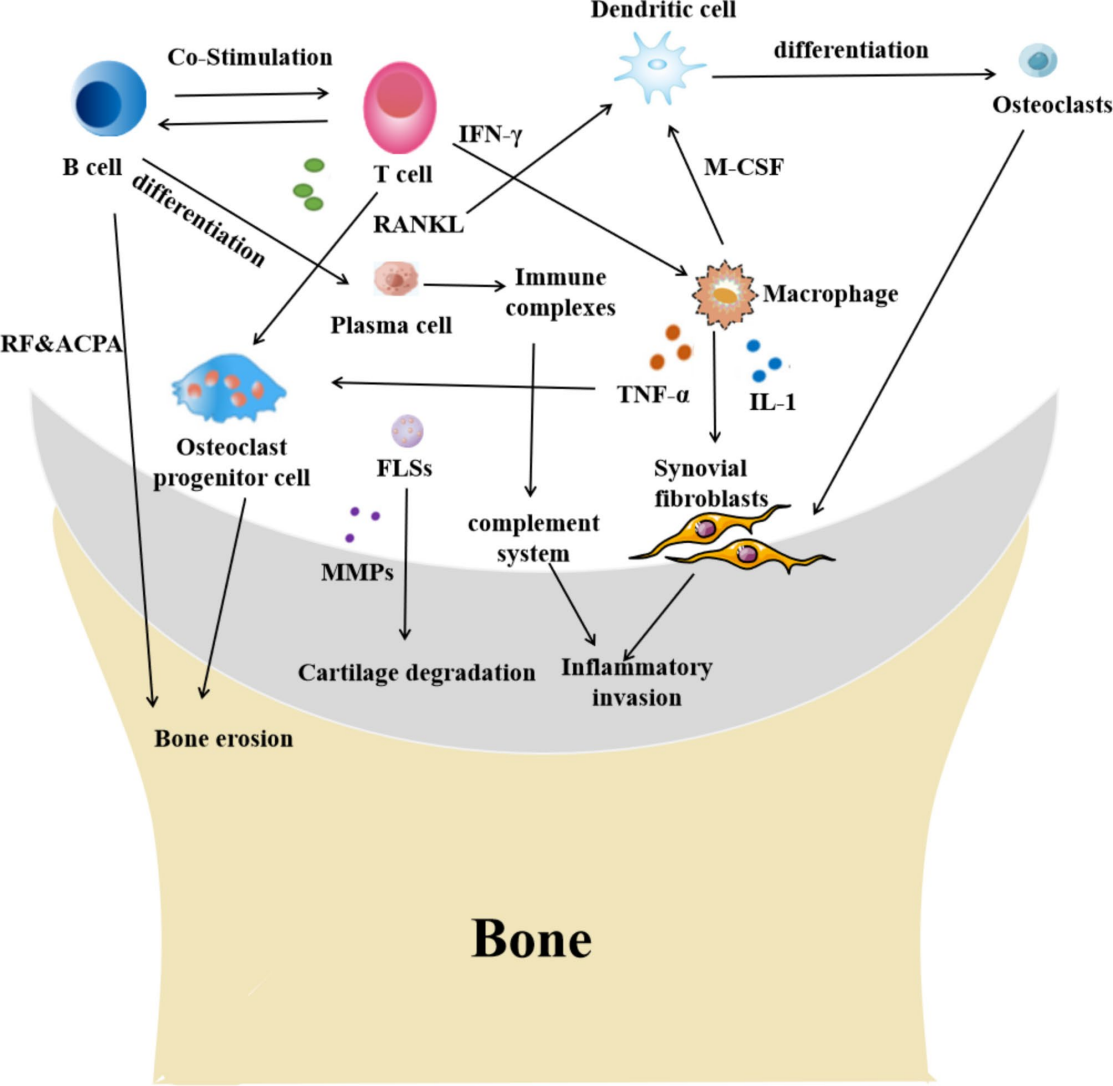
Pathogenesis of rheumatoid arthritis. The occurrence of rheumatoid arthritis (RA) is attributed to the activation of immune cells such as T cells, B cells, macrophages, and dendritic cells. B cells release rheumatoid factor (RF), and dendritic cells differentiate into osteoclasts, leading to bone erosion. T cells secrete receptor activator for nuclear factor-κ B ligand (RANKL) and activate osteoclasts, resulting in cartilage destruction. The overproduction of matrix metalloproteinases (MMPs) by fibroblast-like synoviocytes (FLSs) is also a critical factor in cartilage damage. Excessive immune complex activates the complement system and mediates the invasion process of inflammation. Additionally, interleukin-1 (IL-1) and tumor necrosis factor-alpha (TNF-α) not only cause the accumulation of inflammation at joints but also systemic inflammation. Abbreviation:TNF: tumor necrosis factor; IL-1: interleukin 1; IL-6: interleukin 6; FLSs: fibroblast-like synoviocytes; MMPS: matrix metalloproteinases; RF: rheumatoid factor; RANKL: receptor activator for nuclear factor-κ B ligand;M-CSF:macrophage-stimulating factor;ACPA:Anti-citrullinated peptide antibodies

Macrophages, which are involved in the body’s nonspecific immunity, produce related inflammatory factors such as IL-1, IL-6, and TNF-α. These cytokines will stimulate FLSs and further activate osteoclasts (OCs), leading to bone damage [9–11]. Reports have shown that receptor activator for nuclear factor-κ B ligand (RANKL) is indispensable in osteoclast differentiation and activation, and the number and activity of osteoclasts are key factors in bone destruction [13, 14]. (Fig. 1)

### Therapeutic approachs

**Fig. 2:**
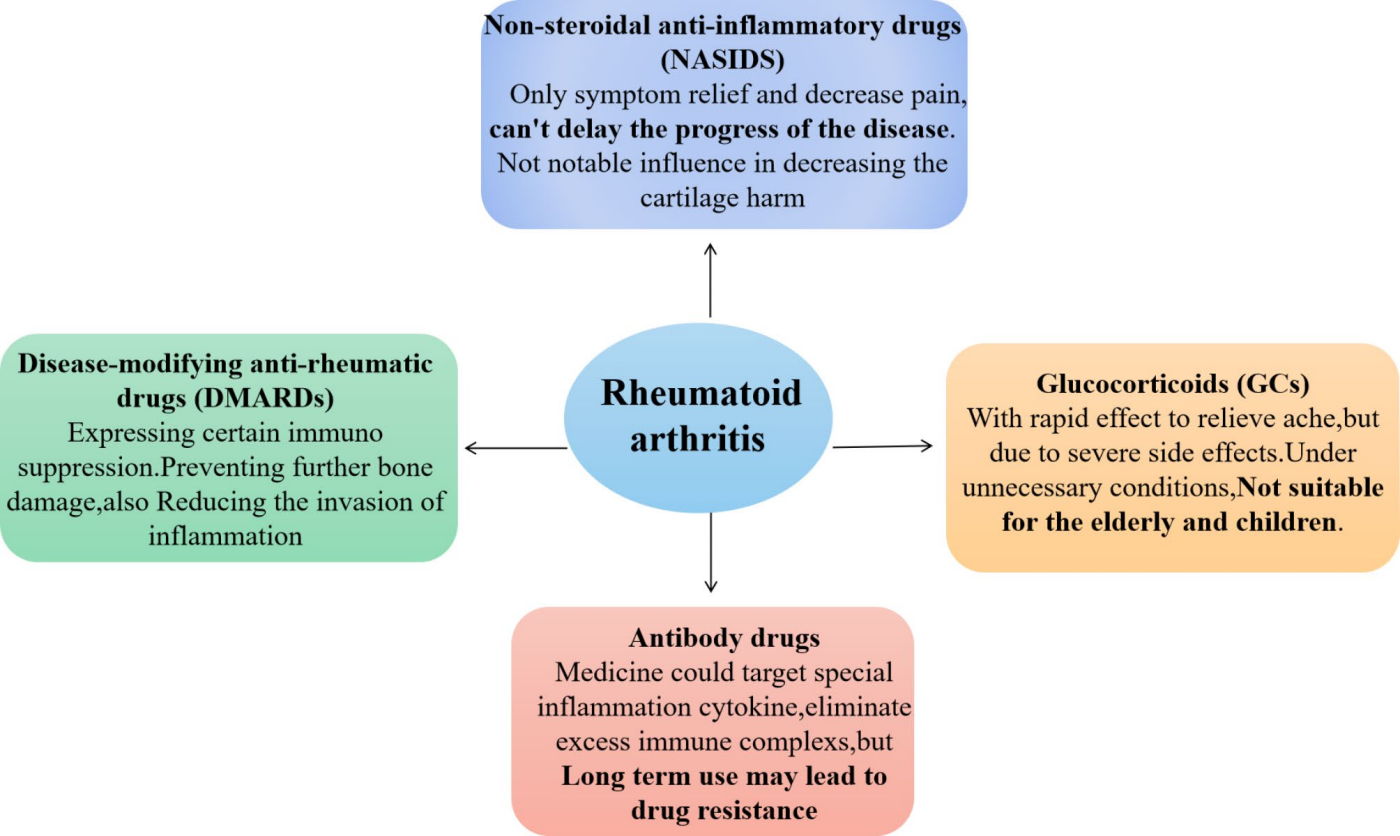
Current drugs used to treat rheumatoid arthritis. There are several treatment strategies available for rheumatoid arthritis, including non-steroidal anti-inflammatory drugs (NSAIDs), glucocorticoids (GCs), disease-modifying anti-rheumatic drugs (DMARDs), and antibody drugs. While NSAIDs can effectively reduce pain in patients, they have no influence on the progress of the disease and do not reduce cartilage damage. Glucocorticoids can rapidly reduce pain, but their usage is limited due to serious side effects, particularly in elderly and pediatric patients. Antibody drugs have clear targets and can reduce inflammation at the lesion, reducing bone and joint injury, and clearing immune complexes and cytokines. However, long-term usage may cause drug tolerance problems. Abbreviation: NASIDS: Non-steroidal anti-inflammatory drugs; GCs: Glucocorticoids; DMARDs: Disease-modifying anti-rheumatic drugs

Clinically, the treatment of RA typically involves surgery and drug therapy aimed at eliminating excessive immune complexes, alleviating pain, delaying inflammation, and maintaining bone and joint function [15, 16]. However, surgery can easily result in secondary joint cavity injury, and removal of the synovial membrane can completely impede synovial fluid synthesis and further compromise joint function. In comparison, drug treatment is less invasive and more versatile, offering an expanding range of options for clinical treatment [17]. The drugs used to treat RA include disease-modifying anti-rheumatic drugs (DMARDs), nonsteroidal anti-inflammatory drugs (NSAIDs), glucocorticoids (GCs), and antibody drugs [18, 25] (Fig. 2). Despite their clinical benefits, DMARDs have poor specificity and low bioavailability, which make it difficult to deliver these drugs to their target in vivo [19]. Furthermore, the toxicity and side effects associated with DMARDs pose a significant risk to patient health. For example, methotrexate (MTX), the most widely used DMARD, can cause ulcerative stomatitis at high doses and liver and kidney damage after long-term use [20]. NSAIDs, although useful as transitional treatment, have limited effectiveness in treating chronic inflammation and carry a high risk of serious side effects [21, 22]. GCs play a critical role in the treatment of inflammatory diseases and exhibit good immunosuppressive effects, but their long-term use raises safety concerns [23]. In contrast, antibody drugs have several advantages, including fewer side effects and better safety profiles, superior efficacy, and high specificity for their target sites [24]. Additionally, their biological pharmacological mechanism is clear, and they have broad applications in treating RA.

Antibody drugs can fundamentally eliminate high-level immune complexes, inhibit inflammatory factors, and alleviate damage to cartilage and synovium, which effectively improve symptoms and relieve pain. Consequently, antibody drugs represent a safe, reliable, and effective treatment method for RA.

## Antibody drugs for the treatment of RA

With the continuous development of molecular biology, we have gained a deeper understanding of the pathogenesis and etiology of RA. In addition, the continuous progress and maturity of biochemistry and nanotechnology have greatly increased the potential application of antibody drugs. Currently, approved antibody drugs are classified into several types based on their mechanisms of action, including TNF-α inhibitors, interleukin 1 inhibitors, interleukin 6 inhibitors, CD80/86-CD 28 inhibitors, and B-cell eliminating antibodies. Antibody drugs that are used for the treatment of RA are shown in Table 1.

**Table 1:** Common antibody drugs used for RA therapy

Drug names	Functions	Outcome	Side effect	Reference
Infliximab	Targeting TNF	Anti-inflammatory properties	Infection, tuberculosis	[34]
Etanercept				[34]
Adalimumab	Targeting TNF	Anti-inflammatory Properties	Infection, tuberculosis	[36]
Certolizumab				[38]
Golimumab				[39]
Anakinra	Combining with IL-1receptor	Reducing IL-1 level	Infection	[43]
Tocilizumab	Targeting IL-6 or IL-6 receptor	Reducing IL-6 level and inflammation	Infection, gastrointestinal perforation	[53]
Abatacept	Targeting CTLA4	Reducing effector T cells	Infection, malignancy	[66]
Rituximab	Targeting CD20 molecule on B cells	Reducing B-cell count and function	Infection, hypertension	[75]

TNF: tumor necrosis factor; CTLA: cytotoxic T lymphocyte antigen; IL: interleukin; CD: cluster of differentiation

### TNF-α inhibitors

Tumor necrosis factor alpha (TNF-α) is a proinflammatory cytokine produced primarily by activated macrophages, T lymphocytes, and natural killer cells [26]. Initially believed to be solely responsible for causing tumor necrosis, TNF-α was later found to be a pathological component of autoimmune diseases and play an essential role in the etiology of RA. Abnormal production of TNF-α mediates synovial hyperplasia and generates other proinflammatory factors, such as prostaglandin (PG) and matrix metalloproteinases (MMPS) [31]. In addition, TNF-α can stimulate bone cells to secrete the receptor activator for nuclear factor-κ B ligand (RANKL), indirectly promoting the formation of osteoclasts, and synergize with various factors to induce RA [27, 28]. Therefore, a broad consensus has been reached to alleviate the disease by eliminating the abundant TNF-α in the inflammatory site. TNF-α inhibitors are the most widely used biological drugs to treat RA. Currently, five TNF-α inhibitors have been approved worldwide for the treatment of RA, including etanercept, infliximab, adalimumab, certolizumab, and golimumab [29]. Although all anti-TNF-α drugs can competitively bind to TNF-α receptors on the cell surface, inhibiting TNF-α biological activity and blocking TNF-mediated cellular responses, the drugs differ in their molecular structures and administration schemes [30].

Etanercept was the first TNF-α inhibitor discovered and the first specific anti-cytokine therapy developed for RA. Its clinical efficacy and safety have been confirmed in early clinical trials [31]. As a TNF-α blocker, etanercept not only inhibits tumor necrosis but also has FC effector activity, which can induce antibody-dependent cell-mediated cytotoxicity (ADCC) and trigger the complement pathway to produce complement-dependent cytotoxicity (CDC) and target immune cell apoptosis [32]. Studies have shown that in cases where patients with RA are inadequate responders to MTX, combining MTX with etanercept leads to significant improvement [33]. In addition, before etanercept was used, immunomodulators were utilized to effectively inhibit antibody production and reduce immunogenicity. However, of particular note, long-term injection of etanercept may induce targeted toxicity, including severe infection, and increase the risk of malignancy and tuberculosis during the course of RA treatment [34].

Infliximab is a chimeric monoclonal antibody that binds to the FC region of human immunoglobulin G 1 (IgG1) and contains a variable region (Fab) of mouse anti-TNF-α. Infliximab can bind to both free types and TNF-α on the cell membrane and competitively inhibit the binding of cytokines and related receptors, with its ADC effect and CDC effect superior to those of etanercept [35].

Adalimumab is the first fully humanized TNF-α monoclonal antibody and the third TNF-α inhibitor after etanercept and infliximab. The Fab fragment of adalimumab binds to TNF-α through a large, highly complementary, strong and stable interface (including the formation of hydrogen bonds and salt bridges) [36]. Adalimumab can also mediate the ADCC effect and exhibits good tolerance and effectiveness in treating RA.

Certolizumab is a pegylated recombinant Fab fragment of a humanized anti-TNF-α monoclonal antibody that binds TNF-α with high affinity and selectivity. Unlike other anti-TNF-α drugs, certolizumab lacks a crystallizable IgG fragment (FC) region and therefore does not mediate CDC and ADCC in vitro. Moreover, certolizumab does not induce granulocyte degranulation or apoptosis of peripheral blood lymphocytes and monocytes in vitro, but it appears to induce the death of nonapoptotic cells [37]. Common adverse reactions related to certolizumab include upper respiratory tract infections, rashes, and urinary tract infections [38].

Golimumab, a human IgG1 monoclonal antibody, is produced by mouse hybridoma cell lines using recombinant techniques and functions by targeting and neutralizing TNF-α. After a single subcutaneous injection, the average time to reach the maximum plasma concentration was 2–7 days. After 12 weeks of injection, a steady-state concentration of blood is reached, with an average absolute bioavailability of approximately 50%. Despite good therapeutic outcome via alone application, its effect is better when combined with MTX. When combined with MTX, the average steady-state valley concentration of the drug is approximately 30% higher than that of patients taking golimumab alone, and the apparent plot ratio is approximately 35% lower [39]. Relevant studies have shown that obese patients exhibit decreased sensitivity to the efficacy of TNF-α inhibitors and have poor efficacy [40].

### Interleukin 1 inhibitor

Interleukin-1 (IL-1) is not a single cytokine but a group of cytokines that can exert local or systemic effects, and it is associated with inflammation and the innate immune response [41]. Furthermore, cytokines from different IL-1 families exhibit diverse physiological effects. For example, cytokines such as IL-1α, IL-1β, and IL-33 exhibit proinflammatory activity, whereas cytokines such as IL-1Ra, IL-37, and IL-38 demonstrate anti-inflammatory activity [42]. Generally, IL-1 and IL-1Ra are in a relatively dynamic balance in healthy organisms to maintain normal vital activities. However, when the amount of IL-1 in the body increases, this balance is disrupted, resulting in inflammation and disease. Therefore, IL-1-mediated proinflammatory responses can be inhibited by blocking IL-1 signaling, which involves competitively inhibiting the specific binding of IL-1 to its receptor by IL-1 receptor antagonists.

Anakinra is a recombinant human IL-1 receptor antagonist with a short half-life and must be injected subcutaneously daily. Common adverse reactions to anakinra include local injection infection, high-dose infection, and immunogenicity [43]. Compared to TNF-α inhibitors, anakinra is less effective in treating RA and is therefore not a primary treatment for RA [44]. However, anakinra has shown promise in treating other diseases, and recent studies have demonstrated its efficacy in treating recurrent pericarditis and adult-onset Still’s disease [45–48].

### Interleukin 6 inhibitor

Interleukin-6 (IL-6) is a multifunctional cytokine that is secreted by various cells, such as B cells, T cells, and phagocytes, and specifically binds to corresponding receptors to exert biological effects. IL-6 participates in numerous biological processes and plays a significant role in pathological processes such as synovitis, bone erosion, and inflammation, including B-cell proliferation, antibody production, and T-cell differentiation. IL-6 also promotes hepatocytes to produce acute-phase proteins, induce leukocytosis and angiogenesis, and activates synovial fibroblasts to express matrix metalloproteinase, causing cartilage damage [49, 50]. Patients with RA usually have elevated IL-6 levels. Therefore, IL-6 is also considered a key cytokine in the pathogenesis of RA.

Currently, IL-6 inhibitors can be divided into the two categories based on their different targets: (1) specifically bind to IL-6 receptors to block IL-6 signaling, including tocilizumab and sarilumab, (2) directly bind to IL-6 to perform a function, including sirukumab and olokizumab.

Tocilizumab is the first IL-6 inhibitor discovered and can be administered intravenously or subcutaneously as a humanized monoclonal antibody for treating moderate to severe RA [51]. Studies have shown that tocilizumab reduces IL-6 levels in the body and lowers the level of cyclic citrullinated peptide antibodies (CCPs) [52]. Moreover, tocilizumab’s cardiovascular risk is not significantly different from abatacept or etanercept [53, 54]. The most common adverse effects of tocilizumab are skin infections, neutropenia, thrombocytopenia, and dyslipidemia[55, 56].

Salizumab is a humanized monoclonal antibody that binds to the IL-6 receptor to function and is generally administered via subcutaneous injection. Compared to tocilizumab, salizumab exhibits better affinity and a longer half-life [57, 58]. Furthermore, salizumab is more effective in improving body function than adalimumab. Besides, salizumab may be useful in patients who are not sensitive to conventional anti-rheumatoid drugs or who do not respond adequately to TNF-α inhibitors [59–61]. The safety of salizumab is similar to that of tocilizumab, with common adverse reactions including infection, erythema at the injection site, and neutropenia. In addition, to reduce the risk of infection, it is recommended that an active vaccine should be avoided while the drug is being administered[62]. Other studies have proven that salizumab exerts a positive effect in reducing pain, regulating mood, and reducing fatigue in patients[63].

### CD80/86-CD 28 inhibitor

T cells are essential in the immune system, but an excessive T-cell response can contribute to RA. Cluster of differentiation (CD) proteins, which are located on the surface of cell membrane, play a crucial role in T-cell activation. CD4 + T cells are the primary T cells involved in synovial infiltration and inflammation. To activate T cells, two signals are required: specific binding of T-cell receptor to major histocompatibility complex (MHC) molecules on antigen-presenting cells (APC cells) and costimulation signaling (binding of CD80 or CD86 on APC cells to CD28 ligands on the surface of T cells). Costimulation signaling is vital in the T-cell activation process. Cytotoxic T-lymphocyte-associated protein 4 (CTLA 4) is a membrane protein that activates T-cell expression, and its structure is similar to that of CD28. Therefore, CTLA-4 inhibitors can prohibit T-cell activation by competing with CD28 and interfering with its binding to CD80 or CD86 [1].

Abatacept is a whole-human recombinant protein composed of the extracellular domain of CTLA 4 and the Fc portion of IG1. Therefore, abatacept can bind to CD80 and CD86 on the surface of APCs and competitively inhibit CD80 and CD86 costimulatory signals. Abatacept can be used for subcutaneous or intravenous administration (both of which have shown similar safety and efficacy properties). Abatacept exerts beneficial effects on clinical symptoms, structural damage, and physical functioning in patients, including those with inadequate responses to TNF-α inhibitors or those for whom MTX is ineffective. Studies have shown that after 12 months of abatacept treatment (10 mL/kg), serum levels of IL-6, C-reactive protein (CRP), and soluble IL-2 receptors were significantly reduced compared with those in the placebo group, and the proportion of memory B cells was correspondingly reduced. Combination therapy with abatacept and MTX has better efficacy than MTX monotherapy, with comparable safety profiles [64, 67, 68]. However, RA patients with interstitial lung disease (ILD) may face the risk of ILD exacerbation when treated with abatacept in combination with MTX. Therefore, if ILD exacerbation occurs with associated complications, MTX should be discontinued immediately [69]. Abatacept is also well tolerated when used in combination with non-MTX anti-rheumatoid drugs that are chemically modified and exhibit similar clinical efficacy to MTX. Abatacept has no significant adverse effects, and the most common adverse reactions are upper respiratory tract infections, nausea, and headache. Owing to the increased risk of serious infections [27, 65], abatacept should not be used simultaneously with TNF-α inhibitors [70].

### B-Cell depleting antibodies

The overabundance of autoantibodies in the body constitutes a crucial factor in the development of autoimmune diseases. In the past few decades, diminishing the number of B cells and their related antibodies has been considered a crucial approach to treating autoimmune diseases [71, 72]. B cells are intimately associated with the pathogenesis of RA. The antigen presentation of B cells is involved in the autoreactive T-cell activation process, and B cells disorderly undergo apoptosis and secrete an excessive number of pathogenic antibodies (RF, CCP, chemokines, etc.), which form immune complexes. Moreover, activating the complement system eventually leads to cell damage. B cell-related autoantibodies may result in infection through the development of inflammatory cells and stimulation of the apoptosis pathway [1].

CD20 is a B-cell differentiation antigen that exists on the surface of B cells at all stages of development and differentiation but is not expressed on plasma cells. It plays a vital role in the proliferation and differentiation of B cells by regulating the flow of synovial calcium ions. Rituximab (RTX), a chimeric human-mouse monoclonal antibody, binds to the CD20 membrane receptor on the surface of B cells and directly induces B-cell apoptosis, eventually leading to B-cell depletion through host-effect mechanisms, such as mediating ADCC and CDC [73]. The combination of RTX and MTX was superior to MTX and placebo, and there was no significant difference in safety. RTX can be used in patients with moderate to severe RA and is also effective in some patients who do not respond adequately to DMARDs or at least one TNF-α inhibitor [27]. Additionally, regardless of the dosage, when RTX is used for RA, MRI found that at week 24, the imaging progress was significantly reduced [74, 75], which met the 20% improvement criteria of American College of Rheumatology’s (ACR20).

Better treatment results were obtained for patients with RF- and CCP-positive RA when using RTX. Studies have shown that after failure of one TNF-α inhibitor, switching to RTX may be a better approach than switching to another TNF-α inhibitor [75]. The safety of RTX treatment for RA is controllable, and the most common adverse reactions during the first infusion include headache, fever, rash, dyspnea, hypotension, nausea, and mild angioedema. Up to 30%~45% of patients will experience these adverse reactions, but they can be relieved by reducing the rate of drug instillation and taking glucose corticosteroids and antihistamines [16, 78]. Compared with non-RTX drugs, patients using RTX have no additional risk of infection, and there is no significant effect on immunoglobulin levels in patients [76]. Furthermore, long-term use of RTX did not cause significant cumulative side effects and was well-tolerated [77].

## Means to improve the efficacy of monoclonal antibody drugs

**Fig. 3:**
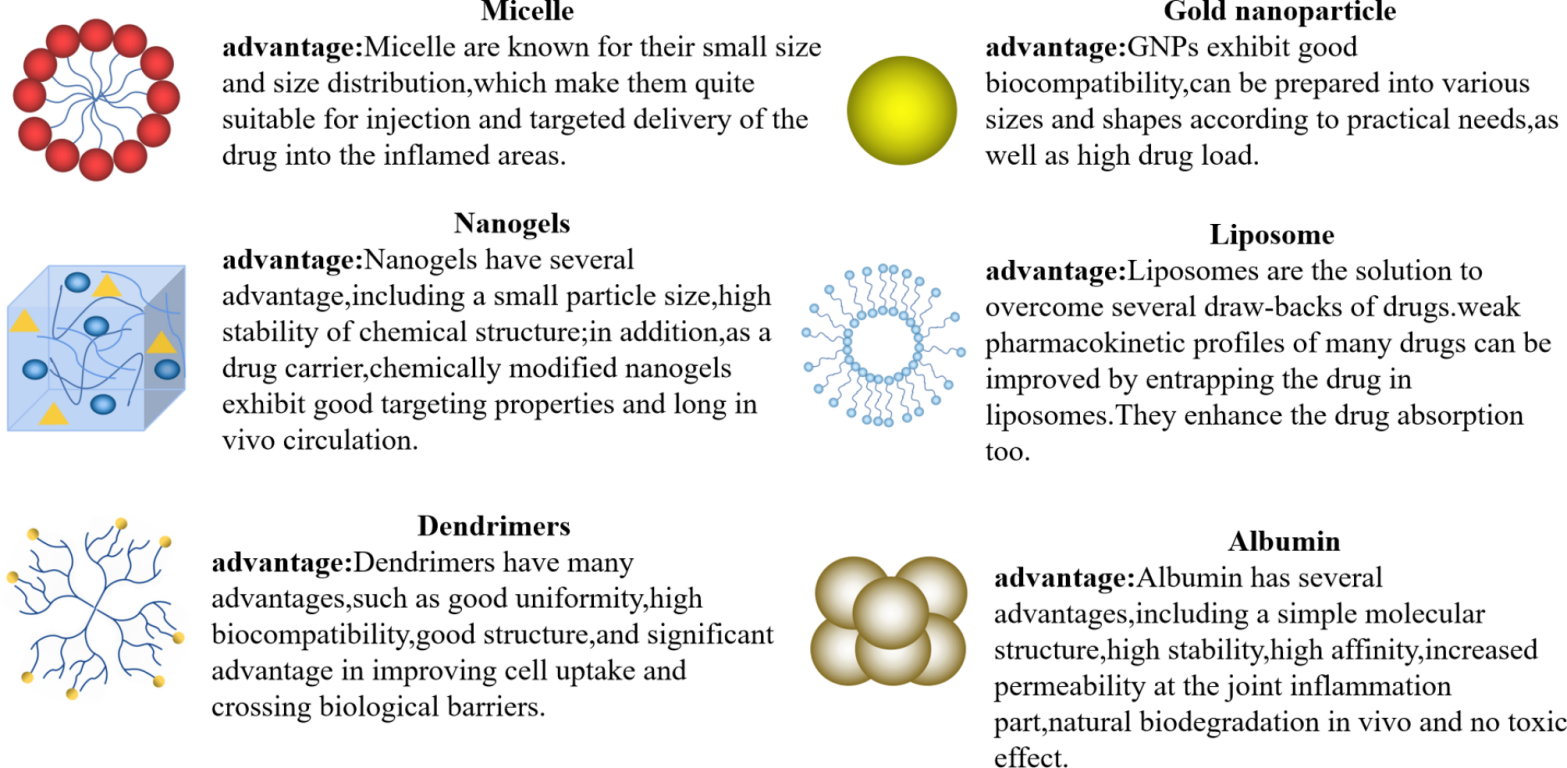
Nanocarriers applied to load drugs. Some nanocarriers can be utilized to overcome the limitations of antibody drugs and enhance their therapeutic efficacy. For instance, micelles are easily to functionalize, which can improve drug targeting. Dendrimers have a large specific surface area, which enhances drug loading capacity and promotes effective drug distribution. Gold nanoparticles are capable of tunable size, photothermal conversion and high biocompatibility, making them useful in combination therapy. Nanogels have revolutionized the way of administration of conventional antibody drugs, since they can be applied directly to the skin surface, thereby improving patient compliance. Albumin, an endonegous protein, dispalys high safety and compatibility. Loading antibody drugs with it can enhance their affinity and reduce adverse reactions

Compared to chemically synthesized drugs, antibody drugs have more explicit targets and lower incidence of severe adverse reactions. However, antibody drugs also exhibit various limitations. For instance, the stability of biological protein drugs may not meet the expected standards, and delivery to target sites in a timely manner can be challenging. Furthermore, many antibody drugs require injection, which can be inconvenient for patients to self-administer. To resolve these problems and improve drug efficacy, different nanocarriers (Fig. 3; Table 2) have garnered increasing attention.

**Table 2:** Recent drug delivery carriers concerning antibody drug

Drug	Carrier/material	Particle size (nm/um) / Zeta potential (mV)	Release behavior	Brief Description	Route	Reference
Etanercept	TMN complex/pullulan-g-oligo(L-lactide)	250 nm/-8mv	TMN complex exhibit slow-release,and the bioavailability is 1.72-fold higher than native etanercept	Improved the long-term stability of etanercept	S.C.	[79]
Etanercept	Microneedle/Hyaluronic acid	-	-	With good biocompatibility and high anti-inflammatory efficacy	Transdermal delivery	[80]
Etanercept	TNG Nanogel/poly(N-isopropylacrylamide)	155.16 ± 22.14 nm/-	approximately 80% cumulative release over the 48 h	EPR	-	[81]
Etanercept	microsphere/methoxypoly(ethylene glycol)-poly(e-caprolactone)-methoxypoly(ethylene glycol)	4.98 ± 0.09 μm/-	At the end of 90-day release study, 98.38 ± 2.11% Etanercept released from MPEG-PCL- MPEG microspheres	Significant decrease in pro-inflammatory cytokines and MMP levels	-	[82]
Etanercept	nanoparticle/PLGA-PEI-mPEG	243 nm/1.0mV	-	EPR	I.V.	[83]
Etanercept	porous three-layer scaffolds/Collagen-chitosan-hydroxyapatite	54 ± 5 nm/-	-	Promote chondrocyte grown and proliferation	-	[84]
Etanercept	nanoparticle/carbopol hydrogel	356 ± 2 nm/-30mv	At physiological- Simulated conditions, with MTX-SLN-ETA releasing about 52 ± 4% in 8 h.	Improved efficacy	-	[85]
Etanercept	nanoflower/molybdenum disulfide	200–300 nm/	In MoS_2_-ETA-PEG,45% of the drug was released after 24 h, and almost 100% was after 144 h.	Inhibited the expression of TNF-α	I.V.	[86]
Infliximab	nanoparticle/polyesterurethane	200-287 nm/−13.93-15.3mv	-	Decreased inflammation cytokine levels	OG	[87]
Infliximab	Conjugated carbosilane dendrimer/carbosilane dendrimer	-	-	-	-	[88]
Infliximab	microparticle/chitosan,carboxymethyl chitosan and alginate	316.5 ± 2.4 nm/−19.6 ± 0.7mv	At pH 6.8, the cumulative release was almost 75% after 8 h.	Avoids the inconvenience of injections and the associated pain	OG	[89]
Infliximab	hydrogel/hyaluronic acid,poly (γ-glutamic acid)	-	66.1% ± 2.0% was demonstrated to be the cumulative released amount of IFX on the 28th day.	Relief pain and protect cartilage	Intra-articular injection	[90]
Infliximab	nanogel/genipin crosslinked fibrin	-	approximately 50% cumulative release over the 20 day	Anti-inflammatory	-	[91]
Infliximab	microsphere/polylactide-co-gly- colide	10 ± 3 μm/-	approximately 65% cumulative release over the 48 h	-	-	[92]
Infliximab	liposome/Aminoclay	406 nm/−55.4mv	approximately 30% cumulative release over the 48 h	Decrease TNF-α level	OG	[93]
Infliximab	Liposome/DSPE-PEG-NH_2_ ,cholesterol	351.3 ± 58 nm/−20.8 ± 9.78mv	accumula-tive drug release reached 70% of total encapsulated infliximab after 7 days	EPR and Anti-inflammatory	Intravitreal injection	[94]
Infliximab	nanoparticle/polyphenol-PEG-containing polymers	100 nm/-20mv	-	Decrease inflammatory level	OG	[95]
Adalimumab	Nanoparticle/ polyester	134 ± 3 nm/-	-	improved the stability and increased side effects	S.C	[96]
Certolizumab	Certolizumab pegol/PEGylation	-	-	Prolonged half-life	S.C	[97]
Anakinra	nanoparticle/folate–chitosan–DNA	110 nm/-	-	Decreased bone damage	I.V.	[98]
Anakinra	Microcapsule/alginate-chitosan	443 ± 36 μm/-	drug released 50.4% in the first 40 min, and the number was above 80% in 120 min.	pH-responsiveness drug release	OG	[99]
Anakinra	Nanoparticle/Block copolymer	300 nm/-	-	Increased the retention time of IL-1Ra	Intra-articular injection	[100]
Anakinra	Microsphere/dextran-PLGA	12.76 ± 4.89 μm/	approximately 80% cumulative release over the 48 h	Prolonged half-life and anti-inflammatory	injection	[101]
Anakinra	Fusion protein/human serum albumin	-	-	Prolonged half-life and delivery drug to inflammatory site	I.V.	[102]
Anakinra	Microparticle/calcium phosphate	-	-	Decrease inflammatory level		[103]
Tocilizumab	nanoparticle/Hyaluronate-gold	60 nm/- 25.65 ± 3.65 mV	-	Dual target and improved efficiency	I.V.	[104]
Tocilizumab	Tocilizumab pegol/PEGylation	-	-	EPR	S.C	[105]
Rituximab	Nanoparticle/gold nanosphere	-	-	Low toxicity and high repeatability		[106]
Rituximab	Liposome/HA-g-DEAP	120–133 nm/−2.7 mV	approximately 50% cumulative release over the 24 h	Improved efficiency	I.V	[107]
Rituximab	liposome/1,2-bis (10,12-tricosadiynoyl)-sn-glycero-3-phosphocholine	317 ± 80 nm/-	approximately 90% cumulative release over the 48 h	Favorable biocompatibility, high serum stability	I.V	[108]
Rituximab	SPION/superparamag- netic iron oxide	140–190 nm/-7.2 ± 0.4mv	-	Cross the blood‒brain barrier	I.V	[109]
Rituximab	RDMN/ 3-(2-Pyridyldithio) propionyl hydrazide	94.1 ± 14.5 nm/-	approximately 90% cumulative release over the 24 h	Showed the higher therapeutic effect	I.V	[110]

ETA: etanercept; IFX: infliximab: RTX: rituximab; ADA: adalimumab; MTX: methotrexate; TMN: temperature-modulated noncovalent interaction; SLN: solid lipid nanoparticle; TNG: thermoresponsive nanogel; MPEG-PCL-MPEG: methoxy polyethylene glycol–polycaprolactone–methoxy polyethylene glycol; PU: polyesterurethane; PEG: polyethylene glycol; PLGA: polylactic-co-glycolic acid; HA: hyaluronic acid.

FibGen: fibrin-genipin; HSA: human serum albumin; NP: nanoparticles; NM: nanoin microparticles; AC-L: Aminoclay-coated liposomes; HA-PGA-IFX: hyaluronic acid-poly(γ-glutamic acid)-infliximab; PPP: PLGA-PEI-mPEG; ACPP: activated cell-penetrating peptides; RDMN: rituximab-doxorubicin micellar nanoparticle; SPIONs: superparamagnetic iron oxide nanoparticles; DEAP: 3-diethylaminopropylamine; PPNP: polyphenol-PEG-containing polymers self-assembled nanoparticles; MoS_2_: molybdenum disulfide; MPS: mineral-coated microparticles; EPR: enhanced permeability and retention; SC: subcutaneous injection; IV: intravenous injection; OG: oral gavage.

### Gold nanoparticles (GNPs)

**Fig. 4:**
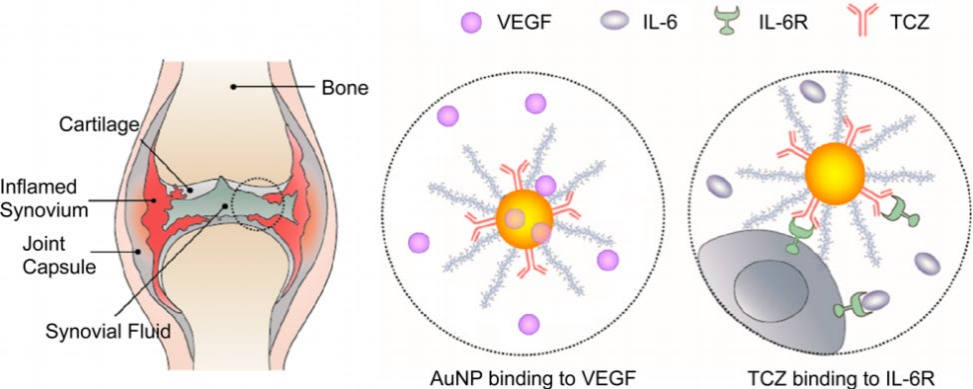
HA-GNP-TCZ targets both VEGF and IL-6R [104].Reprinted from Lee H, Lee MY, Bhang SH, et al. Hyaluronate-gold nanoparticle/tocilizumab complex for the treatment of rheumatoid arthritis. The dual-targeted HA-GNP-TCZ complex was developed to simul-taneously bind VEGF and IL-6R to treat RA. The combination between AuNP and VEGF demonstrated brilliant antiangiogenic effect on RA. TCZ, an immunosuppressive drug, interferes with IL-6 during the pathogenesis of RA. Hyaluronic acid is widely used for cartilage protection and lubrication. This compound alleviates the immune disorder at the joint, and ultimately achieves the therapeutic response of reduced excessive cytokines and repaired cartilage. Abbreviation:TCZ: Tocilizumab ; VEGF:vascular endothelial growth factor; AuNPs:Gold nanoparticles; IL-6:interleukin 6.ACS Nano. 2014;8(5):4790–4798. https://creativecommons.org/licenses/by/4.0/0.44.

In contrast to conventional nanoparticles, gold nanoparticles (GNPs) are increasingly favored by researchers for their effectiveness and stability in drug delivery. GNPs exhibit good biocompatibility, can be prepared in various sizes and shapes according to practical needs, are relatively easy to obtain, and exhibit modifiable and adjustable optical properties, as well as a high drug loading capability. In addition, their noncytotoxicity and lack of serious side effects make them safe for use. In short, due to their excellent performance, the application of GNPs in the biomedical field holds great potential [111–113]. For example, high-atomic-number GNPs can preferentially absorb X-rays to enhance the effect of radiation therapy. In addition, GNPs can be used as nanoprobes and contrast agents for diagnosing RA [113, 114]. GNPs not only exhibit good targeting performance but also have a positive effect on treatment. Studies have shown that GNPs can be combined with vascular endothelial growth factor (VEGF) to exhibit anti-angiogenic effects, which is the maor pathological condition of RA. Zeng et al. [112] found that GNPs are also an important antioxidant that promotes osteogenesis and stem cell proliferation, inhibits RANKL-induced osteoclast production, reduces inflammation levels, and reduces bone erosion or cartilage destruction. Lee H et al. [104] designed a new hyaluronic acid-GNP-tocilizumab (termed as HA-GNP-TCZ) drug delivery systems Firstly, HA was modified with cystamine via reductive amination to synthesize end-group thiolated HA. AuNPs were prepared by reducing and stabilizing HAuCl4 with sodium citrate under boiling conditions. The binding of tocilizumab and thiolated HA onto GNPs increases the stability of GNPs and reduces specific binding to serum proteins in vivo. Most importantly, HA-GNP-TCZ can target both IL-6 and VEGF (Fig. 4). After HA-GNP-TCZ treatment, the level of inflammatory cell infiltration, cartilage destruction and bone erosion decreased significantly. Especially, the interface between cartilage and bone was similar to that of the normal control group. It is noteworthy that no synovial hypertrophy was observed in the HA-GNP-TCZ complex treatment group, in contrast to the synovial hypertrophy with cell infiltration in the TCZ treatment group. Besides, the expression levels of IL-6 and CD68 were significantly decreased after treatment with the HA-GNP-TCZ complex, while they were significantly increased in the negative control group. Shahen et al. [115] showed that targeted treatment of TCZ delivered by GNPs alleviated the narrowing of the joint space and bone erosion, as well as the inflammation. Although GNPs accumulate in different organs, they do not cause any toxicity or cell damage and inhibit the expression of inflammation and angiogenesis mediators, effectively delaying the progression of RA.

### Albumin nanoparticles

Albumin is mainly produced by hepatocytes and is the most abundant protein in human plasma, accounting for approximately 50% of the total plasma protein. It has important physiological functions in vivo, such as maintaining the stability of plasma osmotic pressure, ensuring communication between intracellular fluid, extracellular fluid and tissue fluid. Additionally, it serves as a natural detoxifier by binding to heavy metal ions in the body. Albumin also acts as an important nutrient. It possesses several advantages, including a simple molecular structure, high stability, high affinity, increased permeability at the joint inflammation site, natural biodegradation in vivo and no toxic effect [111].

Furthermore, studies by Liu et al. [118] confirmed that the level of the secreted protein acidic and rich in cysteine (SPARC) in inflammatory joints increases with the invasion of inflammatory cells, angiogenesis, and bone erosion. SPARC has a high affinity for albumin, and its overexpression contributes to the active targeting of albumin nanoparticles. Due to the increased synovial metabolism in RA patients, the joints require more energy and nitrogen sources, thus increasing the demand for albumin. This characteristic facilitates the targeting ability of albumin nanoparticles [116–119]. In summary, albumin is an effective drug carrier that can deliver drugs to the inflammatory joints, prolong the duration of drug action, and improve pharmacokinetic properties and efficacy.

Liu et al. [102] developed a recombinant protein by fusing human serum albumin (HSA) to the carboxyl terminus of IL-1ra, which was produced in Pichia pastoris. The direct fusion of albumin with IL-1Ra had a positive effect, and the fusion protein retained the biological activity of IL-1Ra and exhibited a longer serum half-life. In contrast with the use of IL-1Ra alone, the fusion protein accumulated for an extended period during joint inflammation, with a lower distribution rate in the liver, kidney, lung and other parts, which demonstrated that the fusion protein had excellent targeting performance and a significant therapeutic effect [120].

### Dendrimers

Dendrimers are innovative synthetic polymer with a dendritic structure that have the ability to bind to antibodies due to their large surface structure. They possess massive advantages, such as good uniformity, high biocompatibility, and a well-defined structure that enhaces cellular uptake [111]. The synthesized dendrimers have various skeletons that are directly related to their physico-chemical properties. Common skeletons include polyamidoamine dendrimers (PAMAM), polypropylene imine (PPI), polyesters, and scaffolds containing phosphorus and silicon atoms in the structure [121, 126]. In addition to the internal structure, the peripheral functional groups determine their use. For example, dendrimers with cationic groups are employed as antibacterial agents, and anionic groups are used as antiviral drugs.

The application of dendrimers in the biomedical field has aroused great interest. Nowadays, dendrimers have been used as nanoplatforms for drugs, nucleic acid transporters, contrast agents, etc. [88]. PAMAM is a commercial dendrimer that can be classified into 0–10 generations according to its molecular size. Its surface includes different terminal functional groups, allowing it to covalently attach to the active target molecule. In addition, PAMAM with amine or hydroxyl groups on the surface also exhibits anti-inflammatory activity, making it possible to prepare new drugs [111].

Combining PAMAM with synthetic or natural biodegradable polymers facilitates its interaction with living cells and improves its biological performance. Since chondroitin sulfate (CS) is abundant in tissues, Oliveira et al. [122] modified PAMAM with CS and anti-TNF α antibodies (Abs) to increase the affinity with cartilage. The system can be used for controlled and continuous drug delivery and does not cause harmful effects on the metabolic activity and proliferation of the cells subjected, showing good cytocompatibility and hemocompatibility.

Besides the PAMAM skeleton, the carbon silane skeleton has also attracted considerable attention. According to another study, the carbosilane dendrimer skeleton shows excellent hydrophobicity due to its special structure, which enables it to bind with antibodies and interact with cell membrane more effectively [88].

### Nanogels

Nano-gel is a type of polymeric gel that exists in the form of nanoparticles, with typical network structures of molecular cross-linking. It can disperse into nanoscale hydrogel particles in aqueous solution. Nanogels can respond to different environmental stimuli, such as chemical signal stimulation (pH, chemical or biological substances) and physical signal stimulation (temperature, light intensity, electromagnetic field). Nanogels have several advantages, including a small particle size, high stability of chemical structure, good biocompatibility, good permeability and good water retention. In addition, chemically modified nanogels exhibit good targeting properties and extended circulation time. Thus, nanogels have attracted wide attention in the field of drug carriers [123].

As a topical administration method, nanogels can alleviate adverse reactions caused by systemic administration and reduce the invasive trauma of injection administration, making them more patient-friendly. Studies have proven that nanogels can penetrate the skin and migrate to the epidermis with good permeability. Notably, nanogels possess several characteristics, including high loading capacity for protein drugs, good stability, and controllable protein release, making them ideal carriers for protein drugs [124].

Samah et al. [125] demonstrated that nanogels can effectively deliver drugs to the viable epidermis (VE) and produce a definite anti-inflammatory effect without causing immunogenic or toxic effects. Nguyen et al. [127] reported a new nanogel that depends on agarose-curdlan to load etanercept. The diameter of dispersed nanogel was measured at 30–100 nm. This nanosystem enhanced the permeability and retention effects, assisting in large accumulation at inflammatory sites. Likewise, it protected etanercept from immune clearance and improved its biological half-life.

### Others

Temperature-sensitive drug delivery systems have also gained significant attention from researchers. Jung et al. [79] developed a novel type of temperature-modulated noncovalent (TMN) interaction controllable complex. This TMN complex exhibited mutual electrostatic interactions with positively charged etanercept at a temperature lower than the polymer clouding temperature (CT) of 4 °C. When the temperature reaches physiological conditions (37.5 °C), a new polymer-protein complex is formed by double noncovalent interactions. This significantly improves serum stability and prolongs the pharmacokinetic parameters of etanercept in vivo. Pathological analysis of joint tissue revealed that the TMN complex significantly improved inflammatory cell infiltration without obvious vascular and synovitis formation or cartilage destruction. In addition, bone erosion was alleviated, indicating an augmented therapeutic effect.

**Fig. 5:**
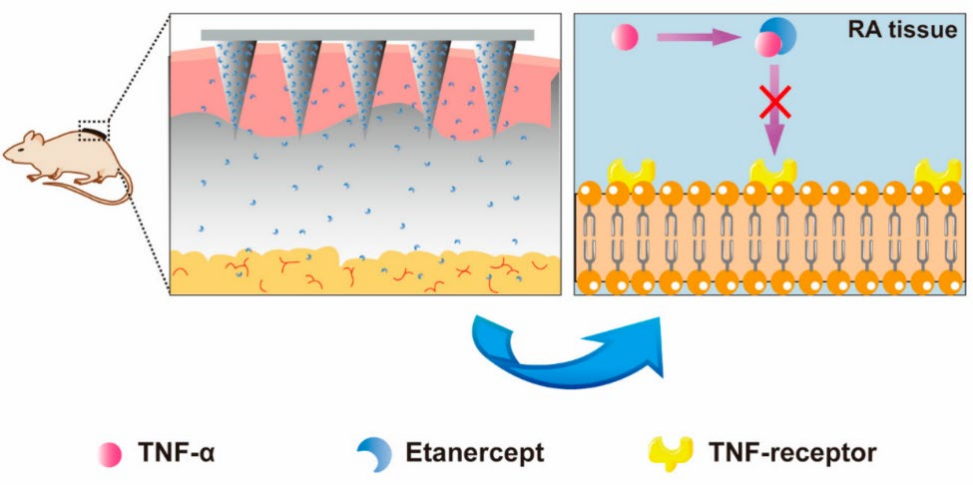
After MNs arrive at the body, etanercept blocks the TNF-α-to-TNF receptor [80]. The microneedle system is applied to the skin on the back of mice, and etanercept (EN) is released from the system and absorbed by the capillaries in the surrounding tissue. In arthritic tissue, EN combines with TNF-α receptors and blocks TNF-α-mediated pathway to exhibit therapeutic potential. Reprinted from Cao J, Zhang N, Wang Z, et al. Microneedle-Assisted Transdermal Delivery of Etanercept for Rheumatoid Arthritis Treatment. Pharmaceutics. 2019;11(5):235.https://creativecommons.org/licenses/by/4.0/0.44.

Since RA is a chronic inflammatory disease, frequent injections may result in discomfort and reduced patient compliance. The emergence of transdermal drug delivery using microneedles has created new opportunities to achieve long-term patient compliance. Cao et al. [80] developed a hyaluronic acid crosslinked microneedle (MN) system as a carrier to deliver etanercept (EN). This system was easy to self-administer after application on the skin, and the drug could be released without additional procedures, reducing the pain from injection. After treatment, the paw swelling ratio of EN treated using MN mice decreased from 1.68 to 1.44 within 10 days, showing a good anti-inflammatory effect. Moreover, the concentration of TNF-α and IL-6 decreased in serum. Pathological sections showed that the joint structure of the saline-treated mice (SA) group was poor. In contrast, EN treated using SC mice (eSC) and eMN effectively protected the joint from erosion. In conclusion, compared with the eSC group, eMN shows similar efficacy in foot swelling, clinical score, cytokines, and joint erosion with classic SC administration. Moreover, MN exhibits higher biocompatibility and compliance, offering great prospects for carrying etanercept (Fig. 5).

## Summary and outlook

Rheumatoid arthritis (RA) is an autoimmune disease that results in the infiltration of inflammatory cytokines and erosion of cartilage and bones, leading to joint swelling, pain, and bone damage, ultimately affecting the survival status and lifespan of patients [130]. Surgery cannot fundamentally solve the excessive immune status of patients, and it causes secondary injury to the patient as the joint synovium is removed. Therefore, drug therapy remains the routine method for the treatment of rheumatoid arthritis. At present, NSAIDs and DMARDs are still used as first-line treatment drugs. However, the lack of clear therapeutic targets, cumulative side effects, and drug resistance caused by long-term use seriously restrict their clinical application [134].

Antibody drugs represent a new choice for the treatment of RA. Antibody drugs have a strong immunosuppressive ability and can clear excessive immune complexes in vivo, contributing to an enhanced therapeutic response [132, 133]. However, there are still some challenges that need to be resolved in their clinical applications. One such challenge is patient compliance as the lifetime maintenance of antibody drugs is generally required. Long-term intravenous administration of antibodies leads to a lack of patient compliance. Therefore, exploring a convenient delivery system for RA treatment is crucial.

The advent of transdermal delivery nanosystems has improved patient compliance by avoiding the inconvenience of injections and the associated pain. Walsh et al. [128] designed nanotopography-based microneedles to enhance the transdermal delivery of etanercept. The nanotopography can combine with integrin to cause tight junction protein remodeling, induce clustering of focal adhesion proteins, and increase paracellular permeability. The prepared microneedles do not penetrate the dermis, thereby alleviating the pain and improving patient compliance caused by intravenous administration. Thus, transdermal drug delivery systems may be a profound way to improve patient compliance for RA patients.

RA is an autoimmune disease. Its pathological manifestations are infiltration of inflammatory cytokines and erosion of cartilage and bones, leading to joint swelling, pain and bone damage, seriously affecting the survival status and life span of patients. Surgery cannot fundamentally solve the excessive immune status of patients but causes secondary injury to the patient because the joint synovium is removed. Therefore, drug therapy is still a routine method for the treatment of rheumatoid arthritis. At present, NSAIDs and DMARDs are still used as first-line treatment drugs. However, the lack of clear therapeutic targets, cumulative side effects, and drug resistance caused by long-term use seriously restrict its clinical application. Antibody drugs represent a new choice for the treatment of RA. Antibody drugs have a strong immunosuppressive ability and can clear excessive immune complexes in vivo, contributing to an enhanced therapeutic response. However, there are still some challenges that need to be resolved in their clinical applications.

1) Patient compliance. Lifetime maintenance of antibody drugs are generally required. Long-term intravenous administration of antibodies leads to a lack of patient compliance. Therefore, exploring a convenient delivery system for RA treatment is crucial. The advent of transdermal delivery nanosystems potentiates improved patient compliance, reflected by avoiding the inconvenience of injections and the associated pain. Walsh et al. [128] designed nanotopography-based microneedles to enhance the transdermal delivery of etanercept. The nanotopography can combine with integrin to cause tight junction protein remodeling, induce clustering of focal adhesion proteins and increase paracellular permeability. The prepared microneedles do not penetrate the dermis; thus, they alleviate the pain and improve patient compliance caused by intravenous administration. Thus, transdermal drug delivery systems may be a profound way to improve patient compliance for RA patients.

2) Immunogenicity. Despite great advances in treatment outcome, antibody drugs still faced secondary failure in extensive patients, featured by adverse events and loss of effectiveness after the secondary application. Emerging evidences indicated that the secondary failure and adverse events were tightly associated with the development of anti-drug antibodies (ADA) in terms of systemic exposure [135]. Chimeric monoclonal antibodies, especially fully human antibodies, were generated to reduce the immunogenicity. Recently, the humanized efficiency was extremely high, up to 99.1% in Golimumab [136]. However, the immunogenicity still cannot be eliminated completely. It was reported that the incidence of ADA from etanercept, golimumab infliximab, adalimumab and certolizumab reach 1.2%, 3.8%, 25.3%, 14.1% and 6.9%, respectively [137]. The occurrence of ADAs may lead to rapid clearance and loss of therapeutic response. Furthermore, PEGylation is wildly used in bioconjugation of antibodies or their fractions to improve solubility and prolong circulation in blood. Whereas, PEGylated antibodies increasingly induce the formation of ADA that specifically recognize and bind to PEG (termed as accelerated blood clearance (ABC) phenomenon). Nanoparticles capable of carrying a payload was regarded as powerful tools to avoid systemic exposure of antigenic epitope in blood circulation, thereby reducing immunogenicity and subsequent clearance [138].

3) Immunosuppression. Systemic exposure of antibody drugs nonspecifically neutralizes cytokines, inhibit antigen presentation or deplete activated B cell, inevitably suppressing the immune system and resulting in potential infection and cancer, especially tuberculosis [34, 36]. Active targeted nanodevices provide a profound strategy to avoid systemic exposure of antibody drugs and aggregate drugs in the lesion. The targeting moiety of existing nanosystems includes a small molecule compound (curdlan) and albumin. It is worth noting that the targeting efficiency of the nanosystems in arthritic joints was not on-demand recently, reflected by the lower fluorescence intensity in paws when compared with that in livers. Increasing evidence indicates that membrane/exosome-coated nanoparticles can significantly improve drug accumulation in the synovium of arthritic joints. Yu et al. [129] prepared hybrid membrane-coated Prussian blue nanoparticles to encapsulate the anti-RA compound schisanlactone E. This multifunctional nanoparticle showed preferential accumulation in paws with respect to that in the liver, creating a possible alternative for improving the biodistribution of antibody-based drugs.

4) Stability. As a protein, the physiochemical properties of antibody drugs can be easily affected by the complicated environment in vitro and in vivo, resulting in increased immunogenicity, reduced half-life and eventually invalidity. Hence, it is critical to stabilize antibody drugs in the storage and administration process. When the antibody drug is encapsulated in the core of nanocarriers (micelles, liposomes, nanocages, vehicles), it can resist external stimuli, contributing to augmented stability. Nevertheless, every coin has two sides. The targeting ability of the antibody itself may be changed without exposure to the microenvironment. Moreover, when antibody drugs are covalently conjugated to the surface of dendrimer/Au nanoparticles, their stability will be influenced by external triggers; however, the targeting ability remains.

5) Target. Majority of antibodies was water-soluble, preventing it from penetrating through cell membrane and interplay with cellular target [139]. Even if internalization into cytoplasm, antibody drugs will be immediately degraded under lysosome conditions with acidic environment (pH 4.0–6.0) and hydrolytic enzymes. To data, various funcational nanodevices have been developped for assisting drugs to escape from endo/lysosomes [140], redirecting down a new way towards effective cellular delivery and further construction of antibody with cellular target.

6) Synergistic effect. Owing to the diminished response over time, monoclonal antibody drugs was suggested to combine with other DMARDs. Increasing evidences revealed that concomitant administration of MTX was invloved in reduced immunogenicity and ADAs [141], thus significantly improved efficacy. Benefiting fron carrying payloads, co-delivery of monoclonal antibody adrugs and other DMARDs can be fabricated in one nanosystem to achieve synergistic effect.

Furthermore, after nanosystems finish the delivery task, the residual nanocarriers can also have a positive effect on the treatment of RA rather than waste. To this end, Zhou et al. [9, 10] prepared tannic acid-based MOF and folic acid-anchored silver nanoparticles. Both of these two nanosystems primarily deliver anti-rheumatic drugs to the joints. Furthermore, the residual nanoplatforms exerted anti-oxidantive effect, pro-apoptosis and re-polarization of macrophages, respectively, contributing to synergistic effect between nanocarriers and drugs.

In addition to functionalize in inflammatory cells, Pandey et al. [131] reported that a hydroxyapatite nanoparticle carrying Teflon and methotrexate successfully transported teriflunomide and methotrexate to the joint, achieving enhanced therapeutic benefits and reduced hepatoxicity. Moreover, the residual hydroxyapatite is a kind of human natural bone component that can promote the proliferation, differentiation and mineralization of osteoblasts. The combination of hydroxyapatite and antibody drugs may not only serve as a good targeted transporter but also play a beneficial role in the process of bone remodeling.

It is a pity that antibody drug loaded nanoparticles barely reached the market. Only some antibody-functionalized nanoparticles have entered clinical trials. For instance, Kadcyla is a nanoscale antibody-drug conjugate (diameter in 15 nm) that consists of the chemotherapy drug DM1 (emtansine) and monoclonal antibody trastuzumab. Clinical trials have shown that Kadcyla can significantly improve the progression-free survival and overall survival of patients with HER2-positive breast cancer compared to other treatments [142]. Kadcyla has also been shown to have fewer side effects than traditional chemotherapy drugs, which can improve patients’ quality of life during treatment. Sgt-94 is an anti-transferrin receptor antibody-engineered liposome encapsulated with a Rb94 plasmid DNA, representing a clinical perspective in phase I in patients with neoplasm (NCT01517464) [143]. Lipovaxin-MM incorporating a specific antibody fragment in the liposomal surface was regarded as a vaccine for malignant melanoma, entering phase I trial.[144]The clinical results demonstrated that Lipovaxin-MM exhibited partial response, well tolerance and absence of severe adverse events .

Accordingly, transformation of antibody drug loaded nanoparticles from laboratory to clinical trials remains an immense challenge, attributing to low drug-loading efficiency, poor reproducibility of nanoparticles, unknown pharmacokinetics property and long-term toxicity [145]. To facilitate the clinical transformation, the property of nanoparticles involved in their performance should be well summarized. Particle size was tightly associated with biodistribution [146, 147], immune response [148] and loading efficiency [149] of monoclonal drugs. Small nanoparticles (less than 10 nm) can rapidly extravasate from inflammatory endothelial windows, while they may be rapidly cleared from the body. Larger nanoparticles (greater than 100 nm) may prolong blood circulation time and achieve desirable accumulation in lesions, but they may be less efficient in penetrating into the deep layers of synovium and cartilage. Similarly, the surface charge of nanoparticles affects their interactions with cells and tissues. Positively charged nanoparticles was favorable to interplay with negatively charged cell membranes and enhance cellular uptake, while they may be hijacked in blood circulation to form “protein crown”. Generally, nanoparticles with a neutral or slightly negative surface charge are preferred for drug delivery to avoid immune recognition and clearance. The drug encapsulation efficiency and release manner can be controlled by adjusting the material properties, size, and surface characteristics of the nanoparticle. Controlled release of the drug can improve drug bioavailability and reduce side effects; however, it may also require more complex nanoparticle designs and manufacturing processes. Therefore, the construction of antibody-based was required to consider a variety of factors comprehensively.

Taken together, as an effective drug to treat rheumatoid arthritis, antibody drugs have broad application prospects and development space in combination with DDSs.

## Data Availability

Not applicable.
